# Effects of Prometryn Exposure on Hepatopancreas Oxidative Stress and Intestinal Flora in *Eriocheir sinensis* (Crustacea: Decapoda)

**DOI:** 10.3390/antiox12081548

**Published:** 2023-08-02

**Authors:** Peng Huang, Liping Cao, Jinliang Du, Jiancao Gao, Yuning Zhang, Yi Sun, Quanjie Li, Zhijuan Nie, Gangchun Xu

**Affiliations:** 1 Wuxi Fisheries College, Nanjing Agricultural University, Wuxi 214081, China; 2021113015@stu.njau.edu.cn (P.H.); caolp@ffrc.cn (L.C.); dujl@ffrc.cn (J.D.); 2020213007@stu.njau.edu.cn (Y.Z.);; 2 Key Laboratory of Integrated Rice-Fish Farming Ecology, Ministry of Agriculture and Rural Affairs, Freshwater Fisheries Research Center, Chinese Academy of Fishery Sciences, Wuxi 214081, China; gaojiancao@ffrc.cn (J.G.);

**Keywords:** triazines, Chinese mitten crab, redox enzyme, inert immunity, inflammatory response, gut microbiome

## Abstract

There is growing evidence that long-term exposure to prometryn (a widely used herbicide) can induce toxicity in bony fish and shrimp. Our previous study demonstrated its 96 h acute toxicity on the crab *Eriocheir sinensis*. However, studies on whether longer exposure to prometryn with a lower dose induces toxicity in *E. sinensis* are scarce. Therefore, we conducted a 20 d exposure experiment to investigate its effects on the hepatopancreas and intestine of *E*. *sinensi*. Prometryn reduce the activities of antioxidant enzymes, increase the level of lipid peroxidation and cause oxidative stress. Moreover, long-term exposure resulted in immune and detoxification fatigue, while short-term exposure to prometryn could upregulate the expression of genes related to immunity, inflammation and detoxification. Prometryn altered the morphological structure of the hepatopancreas (swollen lumen) and intestine (shorter intestinal villi, thinner muscle layer and thicker peritrophic membrane). In addition, prometryn changed the species composition of the intestinal flora. In particular, Bacteroidota and Proteobacteria showed a dose-dependent decrease accompanied by a dose-dependent increase in Firmicutes at the phylum level. At the genus level, all exposure groups significantly increased the abundance of *Zoogloea* and a *Firmicutes* bacterium ZOR0006, but decreased *Shewanella* abundance. Interestingly, Pearson correlation analysis indicated a potential association between differential flora and hepatopancreatic disorder. Phenotypic abundance analysis indicated that changes in the gut flora decreased the intestinal organ’s resistance to stress and increased the potential for opportunistic infection. In summary, our research provides new insights into the prevention and defense strategies in response to external adverse environments and contributes to the sustainable development of *E. sinensis* culture.

## 1. Introduction

Herbicides have greatly improved crop food production and the control of weeds, but they also pose a potential threat to the environment and human health. As the most used class of herbicides, triazine herbicides are widely used in the United States, the European Union and many developing countries including China because of their low price and good weed control effect [1,2]. In 2018, triazine herbicides accounted for 2.1% of the global pesticide use in China, considering that only about 20% of herbicides reach the target organs of plants directly, and the vast majority of the rest affect aquatic habitats directly or indirectly through spray drift, surface water runoff and the consumption of contaminated food, endangering the health of non-target organisms [3,4].

Prometryn, as one of the triazine herbicides, is considered an alternative to atrazine due to its low toxicity [5]. Because of its three nitrogen six-membered ring structures in the molecule, prometryn maintains high stability and consequently has a high half-life and bioconcentration factor [6,7]. However, triazines, including prometryn, are increasingly being detected in oceans, rivers, lakes and even in aquatic organisms with residues, seriously affecting the import and export trade of aquatic products [8,9,10]. Prometryn is the main residue in mariculture areas, accounting for as much as 28% of the total concentration of triazine herbicides in pond-culture and raft-culture areas [11]. In addition, due to its low toxicity to fish, prometryn can be applied directly to aquatic activities for the removal of harmful algae and weeds in aquaculture waters [12]. Therefore, the study of the effects of prometryn on aquatic environment non-target aquatic organisms has also become an important factor in its use in the aquatic environment. A study showed that in aquaculture waters, prometryn presents the highest ecological risk among the investigated agro-veterinary drugs [13]. However, there is a paucity of studies on the negative effects of prometryn on aquatic crustaceans.

*Eriocheir sinensis* is an important freshwater crustacean in China and has a very broad market demand due to its high nutritional value and unique flavor. Its farming production is increasing year by year, and according to the 2022 China Fisheries Yearbook, the production of *E. sinensis* is as high as 8.08 × 10^5^ tons [14]. *E. sinensis* is mainly farmed in ponds, but in recent years a new type of eco-culture for rice crabs has gradually come into view. It should also be noted that prometryn is not only used in pond culture, but also affects aquatic organisms through spraying in paddy fields. Imidacloprid (IMI), an insecticide widely used for pest control in rice fields, could affect the physiological behavior of crustacean aquatic organisms when applied during rice-crab feeding [15]. Previous studies have shown that the commonly used herbicides glyphosate and atrazine are capable of causing oxidative stress, immune response and potential genotoxicity in *E. sinensis* [16,17]. In comparison with insecticides and fungicides, herbicides contribute the most to the total pesticide toxicity in water [18]. Therefore, we hypothesized that prometryn would produce adverse effects in *E. sinensis*, like other herbicides, even at sublethal concentrations.

Several studies have shown that prometryn can cause oxidative stress in aquatic animals, including bony fishes [19,20], molluscs [21] and aquatic crustaceans [22]. Under stressful conditions, the production of reactive oxygen species (ROS) induced by prometryn can cause damage to biomolecules, alter the activity of antioxidant enzymes and also induce inflammatory responses [19]. The immune response is also an important manifestation of exogenous toxicants in crustaceans, which rely mainly on innate immunity due to the lack of an adaptive immune system. The phenoloxidase (PO) system [23], Crustin [24], anti-lipopolysaccharide factors (ALFs) [25], lysozyme (LZM) [26], alkaline phosphatase (AKP) and acid phosphatase (ACP) [27] can be used as important indicators to judge the non-specific immune status of aquatic crustacean animals. On the other hand, neonicotinoid insecticide exposure can alter the activity of drug-metabolizing enzymes in the hepatopancreas (the major organ of drug metabolism) of *Eriocheir sinensis*, most commonly cytochrome P450, which acts as a class of the subtilisin family of proteins involved in phase I detoxification processes [28]. Cytochrome P450 is also considered to be an important and sensitive parameter for the detection of environmental toxicants [29]. After the first phase of detoxification, glutathione-S-transferase (GST) begins to participate in the second phase of detoxification by catalyzing the coupling reaction between glutathione (GSH) and toxic substances, which increases the water solubility of toxic compounds and thus facilitates their excretion from the body, ultimately achieving the detoxification effect [30].

Gut microbes are closely related to the health associations of hosts because the gut is involved in a variety of processes such as the nutrient metabolism, digestion, absorption and immune response of the host [31]. The intestinal flora is exceptionally sensitive to exogenous stresses and when external stresses exceed certain limits, the intestinal flora may change and develop in an unfavorable direction [32]. It has been shown that prometryn disrupts the homeostatic balance of sea cucumber intestinal flora and reduces intestinal microbial diversity [33].

However, there are few studies on the effects of prometryn on the gut flora of aquatic crustaceans, especially *E. sinensis*. Our previous study of acute in vivo and in vitro toxicity has shown that prometryn can cause hepatopancreatic damage through oxidative stress, induce apoptosis, disrupt lipid metabolism, induce immune damage and reduce the proliferative activity of primary cultured hepatopancreatic cells [34]. It is necessary to evaluate the effects of longer exposure to prometryn on *E. sinensis*. In this study, the effects of different concentrations of prometryn long-term exposure on the oxidative stress, immune response, inflammatory reaction, detoxification, histopathology and intestinal flora of *E. sinensis* were investigated. This study contributes to improving the understanding of the pathogenic mechanism of prometryn in aquatic crustaceans.

## 2. Materials and Methods

### 2.1. Experimental Reagents and Animals

Chemicals including prometryn, dimethyl sulfoxide (DMSO), glutamic oxaloacetic transaminase (GOT), glutamic pyruvic transaminase (GPT), alkaline phosphatase (AKP), acid phosphatase (ACP), total protein (TP), catalase (CAT), superoxide dismutase (SOD), malondialdehyde (MDA) and total antioxidant capacity (T-AOC), as well as the reagents used for total RNA extraction, cDNA synthesis and fluorescent quantitative PCR (RT-qPCR), were obtained from commercial suppliers as described in our previous study [34]. Nitrogen monoxide (NO) and GST assay kits were purchased from Nanjing Jiancheng Biological Co., Ltd. (Nanjing, China). LZM and PO were purchased from Shanghai Enzyme-linked Biotechnology Co., Ltd. (Shanghai, China). Then, 150 mg, 300 mg and 600 mg of prometryn were weighed out and dissolved in 15 mL of DMSO to make the master mixes with 10, 20 and 40 mg/L, respectively, for the subsequent experiments. The detection of enzyme activities and other biomarkers followed our previous study [34].

The acclimation, feeding and water quality management of *E. sinensis* followed our previous study [34].

### 2.2. Experimental Design and Sample Collection

Based on the recommended amount of 1 mg/L for the use of prometryn to control hydrilla [33] and the maximum environmental detection concentration of 627.5 μg/L [6], three concentration groups (0.5, 1.0 and 2.0 mg/L) and a control group without prometryn (0 mg/L) were subjected to a 20 d stress experiment. In the following descriptions, these groups are referred to as low concentration (LC), median concentration (MC), high concentration (HC) and control, respectively. Briefly, 1 mL of pre-configured master mix (10, 20 and 40 mg/L in DMSO) was added to 20 L of culture water to configure the exposure group concentration, and 1 mL of DMSO co-solvent was added to 20 L of culture water to configure the control group concentration.

After acclimatization, 180 crabs (19.91 ± 2.88 g) were randomly divided into the 4 groups mentioned above with three replicates each (15 crabs). Taking into account the fact that over 80% of prometryn remains as a residual after 96 h of hydrostatic exposure [33], in this experiment, we changed the solution every 24 h, so that the actual residues of prometryn were not measured. During the exposure period, normal feeding was carried out and water quality conditions were similar to those during the acclimatization period.

Samples were collected at 0 d, 10 d and 20 d, respectively, and nine crabs (three crabs per parallel) from each group were randomly selected for anesthesia on ice for each sampling. The collection of hemolymphs and the hepatopancreas and the latter’s fixation for histology followed our previous study [34]. In addition, at the time of the last sampling, the intestine was additionally extracted for subsequent 16s sequencing and intestinal pathological analysis.

### 2.3. Biochemical Parameters Analysis

Crab hemolymphs were centrifuged (12,000 r/min, 4 °C) for 10 min using a CT14RDII High-Speed Benchtop Centrifuge (Tianmei, Shanghai, China) to obtain serum for the determination of GOT, GPT, AKP, ACP, NO, PO and LZM activities. Approximately 100 mg of hepatopancreatic tissue was mixed with 9 times the amount of saline (0.86%) and was ground on a grinder (Xinzhi, Ningbo, China) (60 Hz, 4 °C) for 60 s, followed by centrifugation (8000 r/min, 4 °C) for 10 min to extract the supernatant for the determination of TP, CAT, SOD, MDA, NO, GST and T-AOC enzyme activities. All procedures were completed according to the instructions of the manufacturer.

### 2.4. Histopathological Observation of Hepatopancreas and Intestinal

Histopathological sections were prepared and observed according to our previous study [34]. A total of six individuals were sampled and fixed in each group, two sections were made for each individual, and three non-contiguous tissue sections were randomly analyzed for each section. Overall, a total of six pathology sections were observed per individual in each group using a light microscope (LEICA DM4 B, Leica, Wetzlar, Germany). The magnification of all sections was approximately 40×.

### 2.5. RT-qPCR Analysis

Total RNA was extracted using the TRIzol method according to the instructions from the manufacturer (Invitrogen, Carlsbad, CA, USA). RNA quality assessment and RT-qPCR condition followed our previous study [34]. All samples were run in triplicate. The primers used are shown in Supplementary Materials Table S1, and the expression of target genes was analyzed according to the 2^−ΔΔCt^ method [35].

### 2.6. 16s Sequencing

Intestinal fecal DNA was extracted using the E.Z.N.A. Soil DNA Kit (D4015 OMEGA, Omega Bio-tek, Norcross, GA, USA) in strict accordance with the instructions. The universal primers 515F (5′-GTGCCAGCMGCCGCGG-3′) and 907R (5′-CCGTCAATTCMTTTRAGTTT-3′) were used to amplify the 16s rRNA gene’s V3-V4 variable region. High-throughput sequencing was performed based on the Illumina MiSeq sequencing platform. The bioinformatics of the gut microbiota were analyzed using the Genepioneer cloud platform (http://112.86.217.82:9919/#/ accessed on 3 February 2023). ASV clustering analysis and species annotation were performed using QIIME 2 (QIIME 2-2021.11) software [36]. Species abundance stacked histogram structural plot analysis, Venn diagram analysis, α-diversity analysis, β-diversity analysis, PLS-DA analysis and phylum-level and genus-level differential species analysis were performed in R (V3.6.2). The prediction of sample phenotypes and the plotting of phenotypic abundance histograms were performed using the R (V3.6.2) BugBase package.

### 2.7. Statistics and Analysis

All data were expressed as means ± SEM. When calculating biochemical and molecular parameters between groups, if the data fit with normal distribution and variance homogeneity, differences measured in different groups at the same time were subjected to one-way ANOVA, followed by Duncan’s multiple interval test in favor of labeled difference letters. Otherwise, we used the nonparametric test (Kruskal–Wallis test), and *p* < 0.05 represented a significant difference. The above data analyses were performed in SPSS (26.0 version, SPSS Inc., Chicago, IL, USA). For gut microbial data analysis, *T*-test analysis was performed between groups using R (V3.6.2) to identify species with significant differences (threshold of significance: *p* < 0.05). “*” indicates a significant difference between the two groups (*p* < 0.05), “**” indicates a highly significant difference between the two groups (*p* < 0.01) as well as if *p* > 0.05 is not shown by default.

## 3. Results

### 3.1. Effect of Prometryn Exposure on Hepatopancreas and Plasma Oxidative Stress Parameters

**Figure 1:**
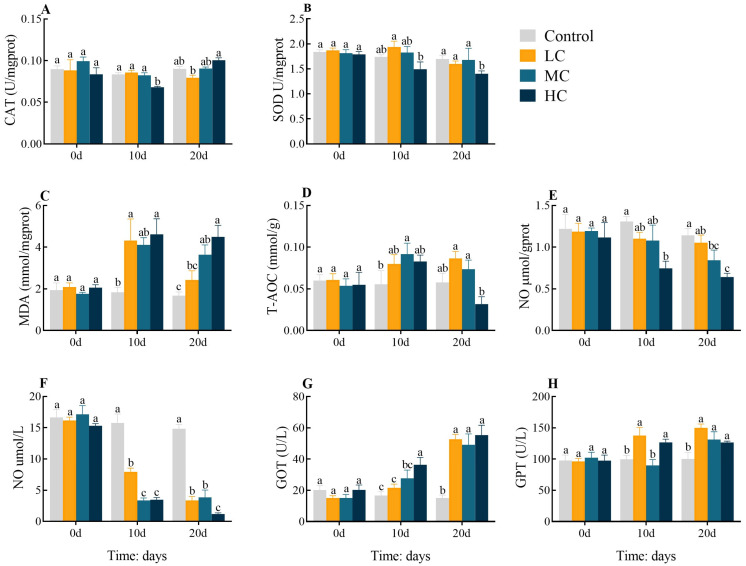
Effect of prometryn exposure on hepatopancreas and serum oxidative stress parameters in *E. sinensis*. (**A**–**E**) and (**F**–**H**) represent hepatopancreatic and serum oxidative stress parameters, respectively. (**A**) Catalase, CAT; (**B**) Superoxide dismutase, SOD; (**C**) Malondialdehyde, MDA; (**D**) Total antioxidant capacity, T-AOC; (**E**,**F**) Nitrogen monoxide, NO; (**G**) Glutamic oxaloacetic transaminase, GOT; (**H**) Glutamic pyruvic transaminase, GPT. Values expressed as means ± SEM (*n* = 9). Data from the control group and the three exposed groups at the same time point were analyzed using one-way ANOVA, followed by Duncan’s multiple-interval test. Different letters indicate significant differences between groups at the same time point (*p* < 0.05).

To investigate the effect of long-term prometryn exposure on the antioxidant status of *E. sinensis*, we measured changes in hepatopancreas and plasma oxidative stress parameters (Figure 1). In comparison with the control, the activity of CAT decreased significantly in HC 10 d (*p* < 0.05, Figure 1A). SOD was lower in HC 20 d (*p* < 0.05, Figure 1B). MDA levels increased dramatically in LC 10 d and HC 10 d. MDA levels were dose-dependently increased and significantly higher in MC 20 d and HC 20 d than in the control (*p* < 0.05, Figure 1C). The T-AOC activity was dramatically raised in MC 10 d (*p* < 0.05, Figure 1D). NO activity showed a dose-dependent decline in both hepatopancreas and serum (*p* < 0.05, Figure 1E,F). The GOT levels showed a dose-dependent rise, with significantly increased levels in HC 10 d compared to the control and significantly increased in 20 d in all exposed groups (*p* < 0.05, Figure 1G). The GPT contents were significantly increased in LC 10 d and HC 10 d. At 20 d, all exposed groups showed a dose-dependent decrease but remained significantly increased compared to the control (*p* < 0.05, Figure 1H). All enzyme activities or levels were found to be largely identical between groups at 0 d (*p* > 0.05).

### 3.2. Effect of Prometryn Exposure on Hepatopancreas and Plasma Immune Parameters

**Figure 2:**
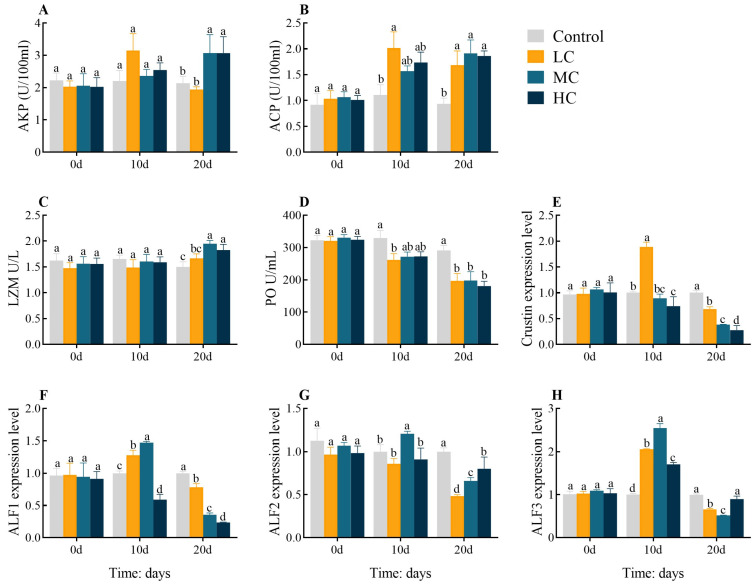
Effect of prometryn exposure on hepatopancreas and serum immune parameters in *E. sinensis*. (**A**–**D**) and (**E**–**H**) represent serum and hepatopancreatic immune parameters, respectively. (**A**) Alkaline phosphatase, AKP; (**B**) Acid phosphatase, ACP; (**C**) Lysozyme, LZM; (**D**) Phenoloxidase, PO; (**E**) Crustin; (**F**) Anti-lipopolysaccharide factor 1, ALF1; (**G**) Anti-lipopolysaccharide factor 2, ALF2; (**H**) Anti-lipopolysaccharide factor 3, ALF3. Values expressed as means ± SEM (*n* = 9). Data from the control group and the three exposed groups at the same time point were analyzed using one-way ANOVA, followed by Duncan’s multiple-interval test. Different letters indicate significant differences between groups at the same time point (*p* < 0.05).

The AKP activities were significantly increased in MC 20 d and HC 20 d compared with the control (*p* < 0.05, Figure 2A). The ACP activities were significantly increased at 20 d in all exposed groups as well as in LC 10 d compared to the control group (*p* < 0.05, Figure 2B). The LZM activities showed significant increases in MC 20 d and HC 20 d compared with the control (*p* < 0.05, Figure 2C). The PO activities were significantly decreased at 20 d in all exposed groups as well as in LC 10 d compared to the control group (*p* < 0.05, Figure 2D). The expression levels of Crustin in hepatopancreas were significantly upregulated in LC 10 d but dramatically downregulated in HC 10 d, and all 20 d exposure groups were significantly downregulated compared to the control (*p* < 0.05, Figure 2E). The expression levels of ALF1 were significantly increased in LC 10 d and MC 10 d, but significantly downregulated in HC 10 d, and significantly downregulated at 20 d in all exposure groups (*p* < 0.05, Figure 2F). The expression levels of ALF2 were significantly upregulated in MC 10 d, while all exposure groups were significantly downregulated at 20 d compared to the control (*p* < 0.05, Figure 2G). The expression levels of ALF3 were significantly upregulated at 10 d in all exposure groups but significantly downregulated in LC 20 d and MC 20 d (*p* < 0.05, Figure 2H). All enzyme activities or gene expression levels were found to be largely identical between groups at 0-d (*p* > 0.05).

### 3.3. Effect of Prometryn Exposure on Hepatopancreas Inflammatory Response and Detoxification Parameters

**Figure 3:**
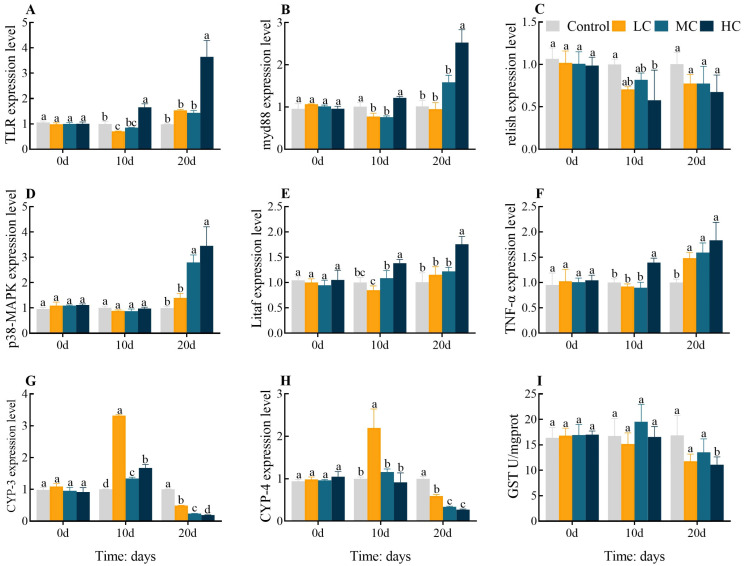
Effect of prometryn exposure on hepatopancreas inflammatory response and detoxification parameters in *E. sinensis*. (**A**) Toll-like receptor, TLR; (**B**) Myeloid differentiation factor 88, myd88; (**C**) Relish; (**D**) p38 mitogen-activated protein kinases, p38-MAPK; (**E**) TNF-alpha factor, Litaf; (**F**) Tumour necrosis factor alpha-like, TNF-α; (**G**) Cytochrome P450 3, CYP-3; (**H**) Cytochrome P450 4, CYP-4; (**I**) Glutathione-S-transferase, GST. Values expressed as means ± SEM (*n* = 9). Data from the control group and the three exposed groups at the same time point were analyzed using one-way ANOVA, followed by Duncan’s multiple-interval test. Different letters indicate significant differences between groups at the same time point (*p* < 0.05).

In comparison with the control, TLR expression levels were dramatically downregulated in LC 10 d but significantly upregulated in HC 10 d and HC 20 d (*p* < 0.05, Figure 3A). Myd88 expression levels were notably downregulated in LC 10 d and MC 10 d, while significantly upregulated in HC 20 d (*p* < 0.05, Figure 3B). The expression level of Relish was significantly downregulated in HC 10 d compared to the control group (*p* < 0.05, Figure 3C). The expression levels of P38-MAPK were significantly upregulated in MC 10 d and HC 10 d (*p* < 0.05, Figure 3D). Litaf expression levels were significantly upregulated in HC 10 d and HC 20 d (*p* < 0.05, Figure 3E). TNF-α expression levels were significantly upregulated in HC 10 d and all 20 d exposure groups (*p* < 0.05, Figure 3F). The expression levels of CYP-3 were dramatically upregulated in all 10 d exposure groups yet significantly downregulated in all 20 d exposure groups (*p* < 0.05, Figure 3G). CYP-4 expression levels were significantly upregulated in LC 10 d but markedly downregulated in all 20 d exposure groups (*p* < 0.05, Figure 3H). The activity of GST was significantly decreased in HC 20 d in comparison with the control (*p* < 0.05, Figure 3I). All gene expression levels or enzyme activities were found to be largely identical between groups at 0 d (*p* > 0.05).

### 3.4. Effect of Prometryn on Morphological Impairment of the Hepatopancreas and Intestine

**Figure 4:**
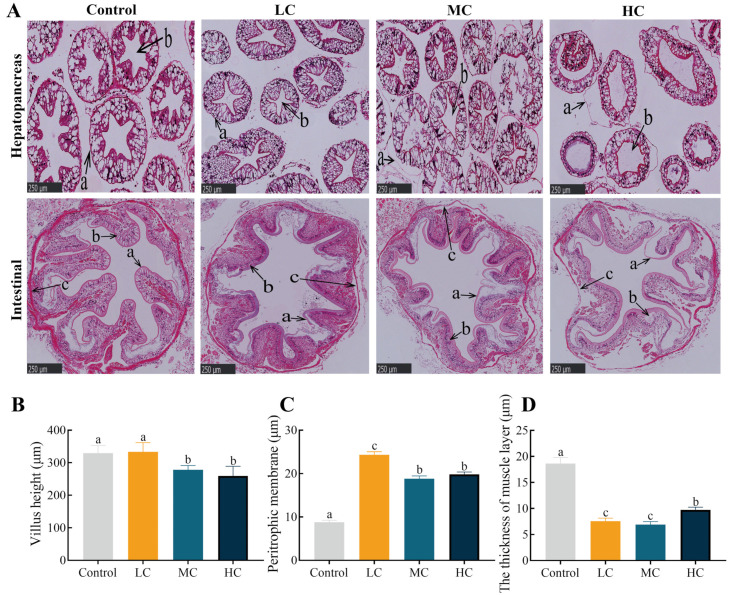
Effect of prometryn exposure on hepatopancreas and intestinal morphology in *E. sinensis*. (**A**) Hepatopancreatic and intestinal pathological variations displayed via hematoxylin-eosin staining. In the first row of hepatopancreas pathology: a, basement membrane; b, lumen. In the second row of intestine pathology: a, intestinal peritrophic membrane; b, epithelial cells; c, muscle layer. (**B**) The villus height of the intestinal tissue. (**C**) The thickness of the peritrophic membrane in the intestinal tissue. (**D**) The thickness of the muscle layer in the intestinal tissue. Values expressed as means ± SEM (*n* = 6). Different letters indicate significant differences between groups (*p* < 0.05).

After prometryn exposure for 20 d, as the dose of prometryn exposure increased, swelling of the lumen and separation of the basement membrane were observed, and this phenomenon was most notable in MC and HC (Figure 4). In the control-group hepatopancreas, the basement membrane was intact, the nuclei were neatly arranged, and the lumen morphology was normal in the stellate shape. In the control intestine, epithelial cells were morphologically distinct and well-arranged. The intestine peritrophic membrane tightly covered the surface of the intestinal lumen. After prometryn exposure, the intestinal villi became shorter and the PM increased in thickness and separated from mucosal fold surfaces (*p* < 0.05, Figure 4A–C). In addition, prometryn significantly reduced the thickness of the intestinal muscle layer and was accompanied by blurring of muscle layer boundaries (*p* < 0.05, Figure 4A,D).

### 3.5. Effect of Prometryn on the Intestinal Microbiota

#### 3.5.1. Community Structures of the Intestinal Microbiota

**Figure 5:**
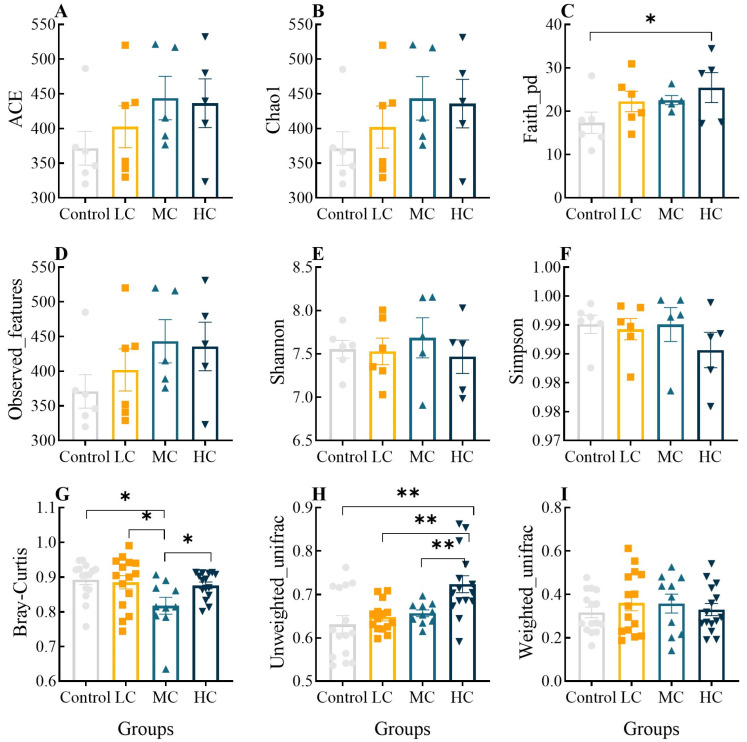
Effect of prometryn exposure on α- and β-diversity indices of intestinal microbial communities in *E. sinensis*. (**A**–**F**) and (**G**–**I**) represent α- and β- diversity indices, respectively. (**A**) Ace diversity index; (**B**) Chao1 diversity index; (**C**) Faith-pd diversity index; (**D**) Overserved features diversity index; (**E**) Shannon diversity index; (**F**) Simpson diversity index; (**G**) Bray-curtis diversity index; (**H**) Unweighted-unifrac diversity index; (**I**) Weighted-unifrac diversity index. Values expressed as means ± SEM (*n* = 6). Bullets, solid boxes and both versions of triangles represent parallel samples within the Control, LC, MC and HC groups, respectively. “*” indicates a significant difference between the two groups (*p* < 0.05), “**” indicates a highly significant difference between the two groups (*p* < 0.01). All data were analyzed using QIIME 2 software and plotted using R (V3.6.2).

A total of 2,006,108 high-quality reads were obtained from 24 intestinal samples in this study via Illumina MiSeq sequencing, and after noise reduction, each sample ranged from 73,539 to 103,436. The good coverage index for each group exceeded 99% and the Shannon index tends to be flat, indicating that the sequencing depth was fully consistent with the community diversity analysis (Figure S1). The α- and β- diversity indices of microbial communities in the control and prometryn-exposed groups are shown in Figure 5. The ace, chao1, overserved features, Shannon, Simpson and weighted unifrac indexes did not vary significantly among groups (*p* > 0.05, Figure 5A,B,D–F,I, respectively). However, the faith pd index of HC was significantly higher than that of the control (*p* < 0.05, Figure 5C), and there was a significant difference in the Bray–Curtis index between the control and MC, between LC and MC and between MC and HC (*p* < 0.05, Figure 5G). The unweighted unifrac index tended to increase in a concentration-dependent manner, and HC was significantly higher than the other three groups (*p* < 0.05, Figure 5H). The results indicate the characteristics of the effect of prometryn exposure on the intestinal microbial community.

**Figure 6:**
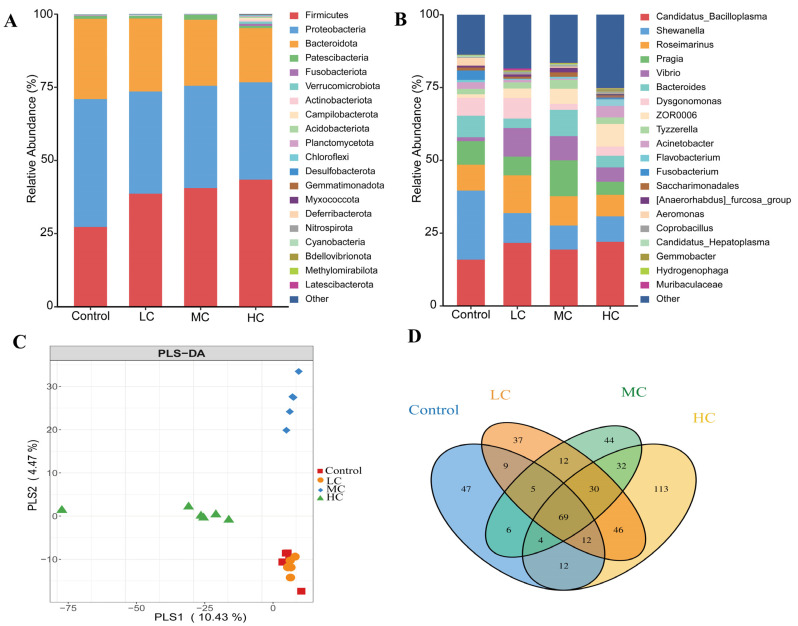
Analysis of the intestinal flora community structure of *E. sinensis* after exposure to prometryn. (**A**) Relative abundance of *E. sinensis* intestinal flora at the phylum level using QIIME 2 software and R (V3.6.2). (**B**) Relative abundance of *E. sinensis* intestinal flora at the genus level using QIIME 2 software and R (V3.6.2). (**C**) PLS-DA analysis based on all samples’ ASV abundance matrix performed using the R (V3.6.2) mixomics package. (**D**) A Venn diagram of species shared and unique for all groups at the genus level using the R (V3.6.2) Venn Diagram package.

There were significant differences in the distribution of intestinal microbiota between the control and prometryn exposed groups (Figure 6A,B). At the phylum level, Firmicutes, Proteobacteria and Bacteroidota are the most abundant phyla of all groups, in which Firmicutes dose-dependently increased, while Proteobacteria and Bacteroidota dose-dependently decreased. At the genus level, *Candidatus_Bacilloplasma*, *Shewanella* and *Roseimarinus* were the dominant genera. Partial least squares-discriminant analysis (PLS-DA) indicates the degree of difference in intestinal flora between the four groups, with the closest distance between the control and LC indicating that they are most similar. In contrast, the distances between MC and the control, and between HC and the control were larger, indicating a greater species difference between them (Figure 6C). The similarity and overlap of OTUs at the genus in different groups are shown in the Venn diagram in Figure 6D. All groups shared 69 genera, and there were 47, 37 and 44 unique genera in the control, LC and MC, respectively. However, there were 113 separate genera in HC, indicating that HC had higher species abundance, which is also evidenced by the diversity index in Figure 5.

#### 3.5.2. Analysis of Significant Differences in Intestinal Flora

**Figure 7:**
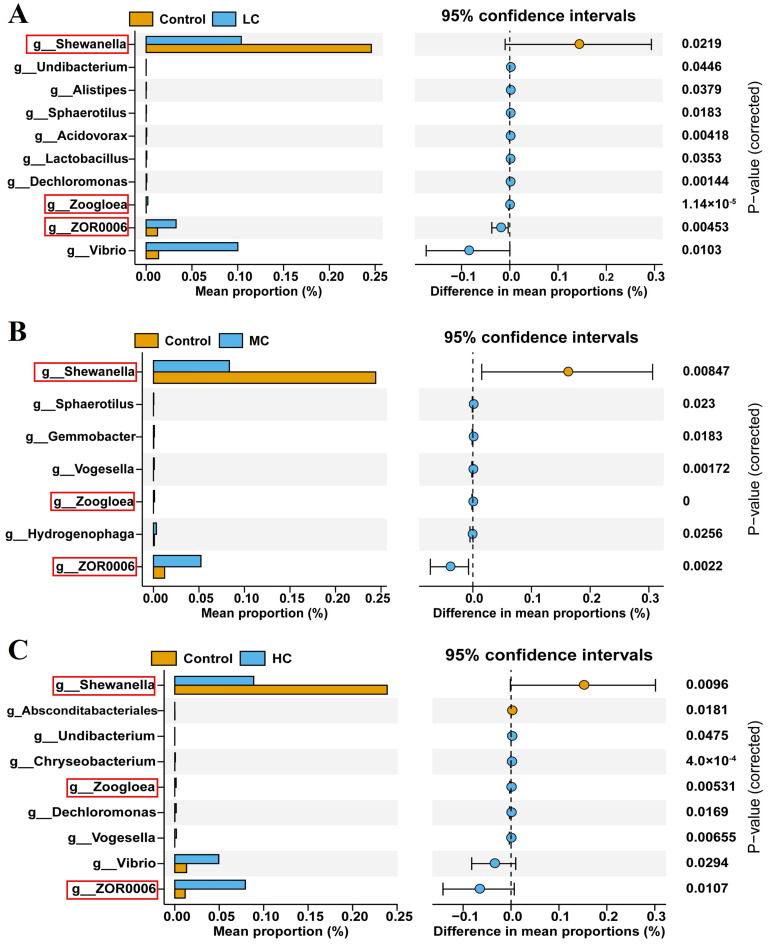
Analysis of intestinal flora on the genus level of *E. sinensis* after exposure to prometryn using Wilcoxon rank-sum test. (**A**) Intestinal flora on genus level between control and LC. (**B**) Intestinal flora on genus level between control and MC. (**C**) Intestinal flora on genus level between control and HC. The three genera with similar abundance differences between the control group and the prometryn-exposed groups (LC, MC and HC) are highlighted by a red rectangle.

When quantifying the differences between the control and exposure to different concentrations of prometryn on the phylum, we found the phyla with significant differences between the control and HC using the *t*-test (Table 1). Bacteroidota, Firmicutes and Proteobacteria were phyla showing significant differences between the control and HC (*p* < 0.05). To further explore genus-level differences in these differentially represented clades, a Wilcoxon rank-sum test showed significant increases in ten genera, six genera and seven genera in LC, MC and MC, respectively. Simultaneously, one genus, one genus and two genera were significantly reduced in LC, MC and MC, respectively. Interestingly, *Shewanella* abundance was significantly reduced, while the abundance of *Zoogloea* and a *Firmicutes* bacterium ZOR0006 was significantly increased in all prometryn exposure groups (*p* < 0.05, Figure 7A–C).

#### 3.5.3. Analysis of Significant Differences in the Phenotypic Abundance among the Intestinal Flora

**Figure 8:**
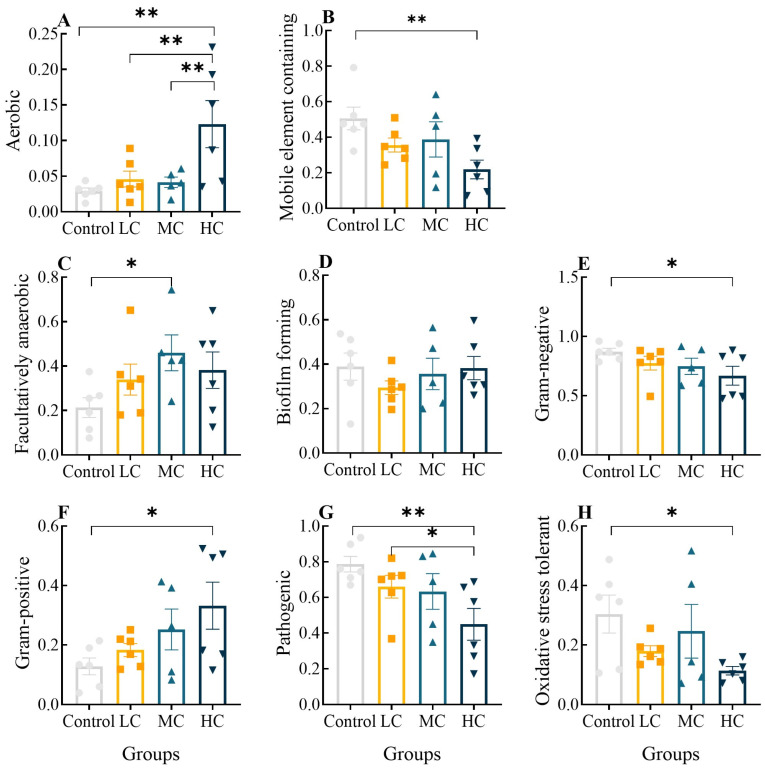
Analysis of significant differences in the phenotypic abundance of the intestinal flora of *E. sinensis* after exposure to prometryn using the Kruskal–Wallis test. (**A**) Aerobic relative abundance; (**B**) Mobile-element-containing relative abundance; (**C**) Facultatively anaerobic relative abundance; (**D**) Biofilm-forming relative abundance; (**E**) Gram-negative relative abundance; (**F**) Gram-positive relative abundance; (**G**) Pathogenic relative abundance; (**H**) Oxidative-stress-tolerant relative abundance. Values expressed as means ± SEM (*n* = 6). Bullets, solid boxes and both versions of triangles represent parallel samples within the Control, LC, MC and HC groups, respectively. “*” indicates a significant difference between the two groups (*p* < 0.05), “**” indicates a highly significant difference between the two groups (*p* < 0.01).

Using Bugbase, we classified microbial communities according to seven phenotypic categories: Oxygen-utilizing (including aerobic and facultatively anaerobic), mobile-element-containing, biofilm-forming, Gram-negative, Gram-positive, pathogenic and oxidative-stress-tolerant communities. The aerobic abundance of intestinal flora gradually rose with increasing exposure concentration, and it was significantly increased in the HC group compared to other groups (Figure 8A, *p* < 0.01). Facultative anaerobics were only significantly elevated in MC compared to the control group (Figure 8C, *p* < 0.05), indicating that high prometryn exposure increased the aerobic respiration of intestinal flora. The abundance of the mobile-element-containing, Gram-negative and oxidative-stress-tolerant phenotypes showed a concentration-dependent decrease and HC was considerably raised compared to the control (*p* < 0.05 or *p* < 0.01, Figure 8B,E,H), whereas the Gram-positive and pathogenic phenotypes showed a concentration-dependent increase and HC was significantly increased compared to the control (*p* < 0.05 or *p* < 0.01, Figure 8F,G). There was no significant difference in the abundance of the biofilm-forming phenotypes between the groups (*p* > 0.05, Figure 8D).

### 3.6. Correlation Analysis of Parameters Related to Differential Intestinal Flora and Hepatopancreatic Injury

**Figure 9:**
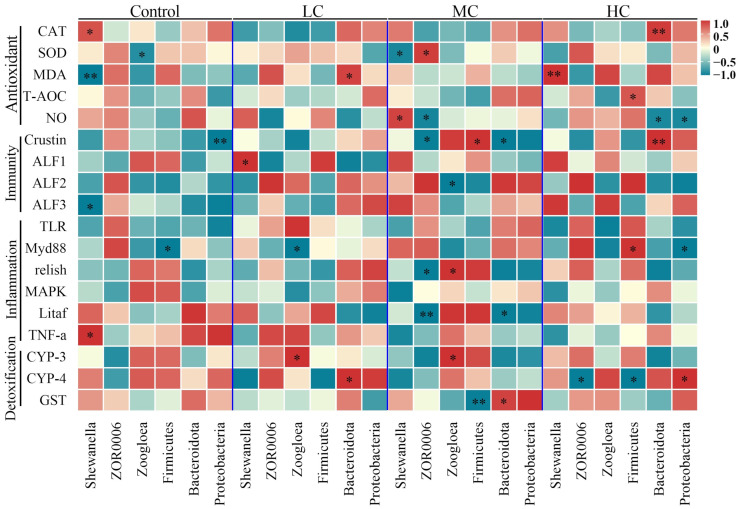
Correlation analysis of differential species of intestinal flora with hepatopancreatic oxidative stress, immunity, inflammation and detoxification-related genes and enzyme activity. Data were subjected to Pearson correlation analysis in SPSS 26.0. “*” indicates significant difference (*p* < 0.05), “**” indicates highly significant difference (*p* < 0.01).

We also established correlations between differential flora in the intestine and parameters related to oxidative stress, immune response, inflammation and detoxification in the hepatopancreas. This better helps us to understand the relationship between differential intestinal flora and hepatopancreatic injury. We found a significant increase in their correlation with increasing exposure dose, especially in MC and HC. In particular, *Shewanella* was mainly associated with oxidative stress in the hepatopancreas, a *Firmicutes* bacterium ZOR0006 was related to oxidative stress and inflammation in MC, and Firmicutes and Bacteroidota were significantly associated with detoxification functions and oxidative stress in the hepatopancreas after prometryn exposure, respectively (Figure 9).

## 4. Discussion

Toxicological studies of herbicides on non-target species are usually associated with oxidative stress, which manifests as an imbalance in oxidation and antioxidation in the body, resulting in oxidative damage. SOD and CAT are the first lines of enzymatic antioxidant systems [37]. Minor yet insignificant fluctuations in these two enzyme activities upon lower-dose prometryn exposure indicates that the free radicals generated by low-dose prometryn exposure could be scavenged by the body’s antioxidant enzymes promptly, thus maintaining normal ROS levels. However, SOD and CAT activities decreased significantly upon higher-dose exposure, which may be related to the body’s oxidative fatigue, where the ROS production rate is much higher than the body’s antioxidant enzyme production rate. A similar study was also seen in *E. sinensis* exposed to low concentrations of avermectin, where CAT and SOD activities were induced, while high concentrations were inhibited [38]. A significant decrease in CAT and SOD was also found in sea cucumbers on diets containing high concentrations of prometryn (2000 mg/kg) for 30 d [21]. Moreover, MDA (a signature product of lipid peroxidation), an important indicator of the level of oxidative stress [39], increased significantly with an increasing dietary prometryn dose [21], which is consistent with our study in which long-term (20 d) exposure to prometryn caused a dose-dependent increase in MDA in *E. sinensis*. In contrast, the short-term surge and recovery of MDA levels at low prometryn doses might be related to the maintenance of the highest levels of T-AOC at low concentrations. *E. sinensis* has developed adaptive mechanisms against prolonged exposure to mild environmental stress. T-AOC is the expression of the body’s total antioxidant capacity, and we found elevated levels of T-AOC at low to medium concentrations of prometryn exposure, as well as a significant increase and then decrease at high-concentration exposure, indicating that *E. sinensis* can enhance its antioxidant capacity to resist oxidative stress at low concentrations. However, exceeding certain concentrations can cause antioxidant fatigue, and hence lead to oxidative damage, as evidenced by the MDA content at high concentrations. NO is a free radical that also serves as an antioxidant, able to react with metal sites and the resulting nitroso complex to inhibit the reaction between the peroxide and the metal, thus preventing the production of ROS [40]. We observed a dose-dependent decrease in NO enzyme activity both in the hepatopancreas and in the plasma. GOT and GPT are sensitive markers of liver injury that are released into the serum when hepatopancreas damage occurs [41], and we found that they increased in a dose-dependent manner. In summary, we infer that short-term low- to medium-concentration prometryn exposure induced alterations in antioxidant enzyme activities and increased the level of antioxidant capacity in the body, while a long-term high concentration inhibits the activities of antioxidant enzymes and reduces the antioxidant capacity of the body, thus causing oxidative damage.

Previous studies have shown that acute (48 h) exposure of shrimps to 0.505 mg/L prometryn significantly altered the expression of genes associated with the immune system [42]. Our previous research has also shown that acute prometryn exposure can alter immune-related sensitivity markers (AKP and ACP) [34]; however, there is a lack of information on the effects of long-term exposure to low concentrations of prometryn on the immune system of *E. sinensis*. PO, as an important component of humoral immunity, is extremely sensitive to the external environment, and even a small amount of exogenous invasion can promote the activation of PO [43]. In the present study, prometryn significantly inhibited PO activity in a time-dependent manner, which again demonstrates that PO is a more sensitive immune indicator to exogenous toxicant stress and can inhibit its activity even at low concentrations with long-term exposure. LSZ is a component of cellular immunity, mainly composed of neutrophils and phagocytes, activating complementary mechanisms and phagocytes of *E. sinensis* to destroy pathogens, and to some extent, AKP and ACP have similar expression patterns to LSZ [44]. The increase in AKP and ACP activities over time under LC conditions, and the dose-dependent increase in LZM activity at the end in this study are consistent with the results of the long-term exposure of *E. sinensis* to microplastics and semicarbazide [44,45]. Therefore, we hypothesize that long-term prometryn exposure would firstly cause damage to the humoral immunity of *E. sinensis* and activate cellular immunity in blood cells and the hepatopancreas. In addition to immune-related enzymes, we also examined the transcript levels of immune-related genes. Crustin and the ALFs family, including ALF1, ALF2 and ALF3, are all important antimicrobial peptides in crustaceans [46]. Significant upregulation of their expression levels in the short term under LC or MC prometryn exposure agreed with the results of acute thiamethoxam exposure [47], suggesting that antimicrobial peptides play a positive role under mild stimulation by herbicides in the short term. However, the expression levels of Crustin and ALFs were significantly downregulated with deeper doses and prolonged exposure, indicating that *E. sinensis* was immunosuppressed by the compound, which is consistent with the results of red swamp crayfish exposed to different concentrations of atrazine (which belongs to the same triazine family of herbicides as prometryn) [48]. Above all, short-term exposure to prometryn can activate the immune system of *E. sinensis*, and long-term exposure disrupts the immune system of the body and subsequently causes immunosuppression.

Prometryn exposure triggered oxidative stress and immune response, which could further induce an inflammatory reaction. TLR (Toll-like receptors) is a pathogen-recognizing molecular pattern receptor that activates MyD88 to promote the expression of NF-ĸB and p38-MAPK signaling pathways [49]. Activation of the p38-MAPK could promote the expression of Litaf and further enhance the expression of tumor necrosis factor alpha (TNF-α) [50,51]. TNF-α is an important pro-inflammatory mediator that facilitates the onset and development of inflammatory reactions [52]. In the present study, short-term (10 d) exposure to low and medium concentrations did not promote the expression of inflammatory factors. In contrast, the expression levels of TLR, myd88 and Relish (NF-ĸB nuclear transcription factor, which has the same potency as NF-ĸB in *E. sinensis* [53,54]) were downregulated, while that of p38-MAPK was not significantly changed. We hypothesize that this is related to the short-term overexpression of relevant immune factors, which are unfavorable to the formation of inflammatory factors. However, with the increase in exposure concentration and the prolongation of time, we found that the above inflammatory factors showed dose-dependent upregulation, except for Relish, which was inhibited. This indicates that prolonged exposure to prometryn can induce inflammatory responses in *E. sinensis*, which may be related to the activation of the p38-MAPK signaling pathway. Similar activation pathways occur in acute salinity exposure and long-term copper stimulation [55,56].

The hepatopancreas is an important digestive and detoxification organ of crustaceans [57]. Therefore, toxic substances are circulated through the hepatopancreas [58]. Previous studies have shown that prometryn can induce the expression of CYP450-related genes in zebrafish [59]. However, the detoxification characteristics of CYP450 in the effect of prometryn on crustaceans are still unclear. CYP-3 and CYP-4 subfamilies have been shown to play important roles in the xenobiotic metabolism of crustaceans [60]. In the present study, short-term exposure to 0.5 mg/L prometryn significantly induced the expression of CYP-3 and CYP-4. However, all exposure groups decreased significantly under long-term exposure. GST, which is the second stage of detoxification, was not activated under short-term exposure, but its activity was also inhibited by long-term exposure. Exposure of T-2 toxin and thiamethoxam to *E. sinensis* provided similar results [47,61]. Preferential activation of the CYP detoxification system, especially CYP-3 and CYP-4, upon prometryn exposure to *E. sinensis*, indicates that low-dose short-term exposure upregulated the expression of CYP-3 and CYP-4 genes due to the self-defense mechanism of crabs. On the other hand, high-dose long-term exposure may have disrupted the detoxification system of the body, thus downregulating the expression of related detoxification genes or enzymes. However, further exploration of the resistance mechanism of *E. sinensis* to the toxicity of prometryn is required.

Continuing excessive levels of oxidative stress can lead to the depletion of the antioxidant defense system, which in turn can lead to damage at the cellular and tissue level [62]. As the main immune and digestive organs of aquatic crustaceans, the hepatopancreas and intestine play important roles in the processes involved in nutrient absorption and metabolism as well as immune defense. All of these require a healthy histomorphological structure and a complete barrier function. In this study, 20 d of exposure to prometryn disrupted the structure of hepatic tubules and caused lumen swelling, which was similar to the results of our previous acute toxicity research [34], again proving the susceptibility of *E. sinensis* to exogenous herbicides. A similar pathology was observed in the hepatopancreas of *E. sinensis* after enrofloxacin exposure [63]. In addition, prometryn exposure significantly altered the structure of the intestinal morphology including shorter intestinal villi and thinner muscle layer, which is an important indicator of intestinal barrier function [64]. Similarly, florfenicol (antibiotics) exposure significantly reduced the intestinal villi and muscle layer of *E. sinensis* [65]. The peritrophic membrane, similar to goblet cells, has the function of digesting and eliminating pathogens and harmful substances [66]. β-conglycinin can damage the peritrophic membrane and separate it from the intestinal folds [67], which is similar to the results of our study. The peritrophic membrane is significantly thickened after prometryn exposure, which may increase intestinal tolerance to exogenous toxicants [68], but further studies are needed. All this evidence suggests that long-term prometryn exposure can alter the morphological structure of the hepatopancreas and intestine and damage the intestinal barrier, which in turn leads to the invasion of opportunistic bacteria.

Herbicides have been increasingly shown to alter gut microbial diversity and gut flora composition in non-target organisms [60,69,70]. In this study, prometryn exposure did not affect the α-diversity index. Aquatic animals themselves have a much greater influence on their gut microbiome than the environment; moreover, the core flora is closely related to the host genotype and is less likely to fluctuate when influenced by the external environment [71,72]. However, significant differences in the Bray–Curtis and unweighted-unifrac indexes in terms of β-diversity, especially a dose-dependent increase in unweighted-unifrac index diversity, indicate that prometryn exposure did not alter the gut microbial richness or evenness, but rather changed the specific type of intestinal flora. At the phylum level, Firmicutes showed a dose-dependent increase, while Bacteroidota and Proteobacteria dose-dependently decreased. A previous study also found a decrease in Proteobacteria abundance accompanied by an increase in Firmicutes after exposure of *Apostichopus japonicus* to prometryn, which is similar to our findings [21]. Increased Firmicutes could contribute to lipid metabolism by increasing the accumulation of fatty acids. A severe imbalance in the ratio of Firmicutes to Bacteroidota could affect the energy metabolism of the host, leading to the invasion of pathogenic microorganisms. This opportunistic infection could in turn affect the health of the host, like in the case of non-alcoholic fatty liver disease. An excess of Firmicutes and a lack of Bacteroidota have been associated with liver disease [73,74,75]. Likewise, a similar phenomenon was found in *E. sinensis* with hepatopancreatic necrosis disease (HPND), with change in the gut microbiome [76]. Proteobacteria is a beneficial bacterium in the intestinal tract of *E. sinensis*, responsible for carbon complexation and denitrification in crustaceans [77]. Therefore, we hypothesize that the decrease in beneficial bacteria and the imbalance in the ratio of Firmicutes to Bacteroidota were influenced by prometryn, which may be further associated with hepatopancreas disease. This was also illustrated via correlation analysis, as the decrease in the abundance of beneficial bacteria (Proteobacteria) and the imbalance in the ratio of Firmicutes and Bacteroidota showed significant associations with parameters related to oxidative stress, detoxification and inflammation in the hepatopancreas, but the mechanisms need to be further investigated.

At the genus level, we found that prometryn significantly increased the abundance of *Zoogloea* and a *Firmicutes* bacterium ZOR0006 but decreased the abundance of *Shewanella* in all exposure groups. *Zoogloea* has been reported to have multifunctional metabolic capabilities, including effects such as influencing the secretion of extracellular polymeric substances (EPS) [78]. The *Firmicutes* bacterium ZOR0006 belongs to the family *Erysipelotrichaceae* and is thought to be associated with metabolic disorders and inflammatory diseases in the host [79,80], which were indeed found to be significantly associated with hepatopancreatic immunity and inflammation in Pearson correlation analysis. *Shewanella* is considered a beneficial bacterium in aquaculture with antimicrobial activity and is commonly used as a probiotic to prevent and treat diseases [81,82]. A previous study also found a significant decrease in the abundance of *Shewanella* in *Penaeus vannamei* under *Vibrio parahaemolyticus* infection [83]. All these results suggest that prometryn exposure increases susceptibility to pathogen infection, as evidenced by the phenotypic abundance analysis of the intestinal flora, with a gradual decrease in the abundance of the oxidative-stress-tolerant phenotype abundance accompanied by a dose-dependent increase in the pathogenic phenotype as the dose of prometryn exposure increases.

## 5. Conclusions

A 20 d long-term toxicity exposure revealed that prometryn induced oxidative stress, immunosuppression, inflammatory responses and detoxification disorder in the hepatopancreas of *E. sinensis* with dose-dependent changes. In addition, long-term exposure to prometryn induced hepatopancreas and intestinal histomorphological damage. Moreover, the exposure to prometryn altered the structure of the intestinal flora of *E. sinensis*, increasing the proportion of harmful bacteria and decreasing the proportion of beneficial bacteria. Therefore, reasonable use of prometryn should be considered in production to avoid negative effects on aquatic animal culture due to environmental damage and pollution.

## Figures and Tables

**Table 1:** Analysis of phyla with significant differences in MC and HC compared to the control using the *t*-test.

Taxonname	Mean Abundance		*p*-Value
Bacteroidota	0.27749 (control)	0.14669 (HC)	0.016
Firmicutes	0.27330 (control)	0.43423 (HC)	0.022
Proteobacteria	0.43711 (control)	0.33298 (HC)	0.049

Control, control group; LC, low-concentration group (0.5 mg/L); MC, median-concentration group (1.0 mg/L); HC, high-concentration group (2.0 mg/L).

## Data Availability

The data are contained within the article and Supplementary Materials.

## Supplementary Materials

The following supporting information can be downloaded at: https://www.mdpi.com/article/10.3390/antiox12081548/s1. Table S1, Primer sequence used for qRT-PCR.; Figure S1, Shannon curves and good coverage index for each group of intestinal flora.
