# Supplementary material for: Effects of Prometryn Exposure on Hepatopancreas Oxidative Stress and Intestinal Flora in Eriocheir sinensis (Crustacea: Decapoda)
Source: Antioxidants (Basel). 2023 Aug 2;12(8):1548. doi: 10.3390/antiox12081548 (PMC10451815; doi:10.3390/antiox12081548)
Supplement: Supplementary file 1 [file antioxidants-12-01548-s001.zip › antioxidants-2515635-supplementary.pdf]

Table S1. Primer sequence used for qRT-PCR.

Category	Gene	Primers	Accession No.
Internal reference	β-actin	F: TCATCACCATCGGCAATGA<br>R: TTGTAAGTGGTCTCGTGGATG	XM_050843218.1
Immunity	Crustin	F: GCTCTATGGCGGAGGATGTCA<br>R: CGGGCTTCAGACCCACTTTAC	OK104808.1
	ALF1	F: GCTGGCTGGACCGGATTATT<br>R: ATCACACGGGTGTTGCAGAT	XM_050871541.1
	ALF2	F: TGTCACCCCGCCTCATTAAG<br>R: GTCAGAGACTCCCCCTGGAT	XM_050871543.1
	ALF3	F: TCTATGGCACAACGACACCG<br>R: TGCCCTCGTGGTACAATTCC	XM_050836286.1
Inflammation	TNF-α	F: GTGGACATCTGGTCAGTGGG<br>R: GGCTCATCTGAGGGATCTGC	AB183467.1
	TLR	F: AGCTTGCCGATTCACACTCA<br>R: CACAGCTCTTCCTCCGTCAG	XM_050878072.1
	Myd88	F: GCCATCGCAGTCGCCAAGTT<br>R: GGCATCCTGTTCATCCAGTTCTGAC	XM_050877740.1
	Relish	F: TCTCCCTACTCTGACCATTCC<br>R: TTCCCACCATCTCACTCTTGT	XM_050843538.1
	Litaf	F: TAAAGGCAAGGGAGGCTTCG<br>R: GAATGGAGCTTGAGGTGGCA	KF892539.1
	P38-MAPK	F: CACTCATGGGTGCTGACCTC<br>R: TACTTGAGGCCTCGCAACAC	JX489772.1
Detoxication	CYP-3	F: CTCCACGACTACAAGATGTTACGC<br>R: CACCTCGTTCATGGTAAAGAGC	XM_050869950.1
	CYP-4	F: AAGACTTCGTGGAGGTGTTC<br>R: GCACAGCGTTATGTTGGTGAAG	XM_050840850.1

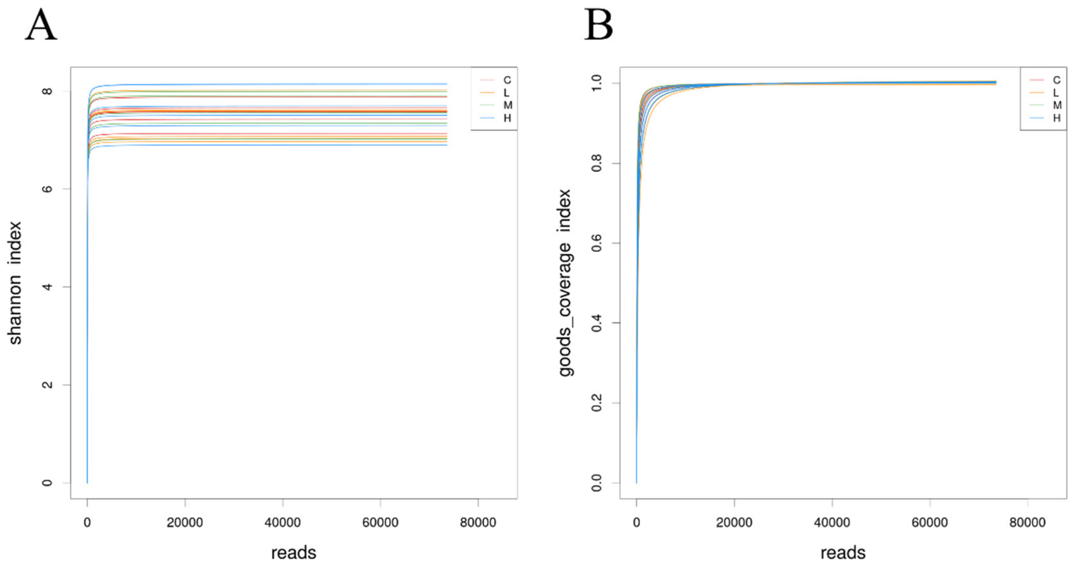

Figure S1. Shannon curves and good coverage index for each group of intestinal flora.
